# A Storage Ring Based Free-Electron Laser for Generating Ultrashort Coherent EUV and X-ray Radiation

**DOI:** 10.1038/s41598-017-04962-5

**Published:** 2017-07-05

**Authors:** Chao Feng, Zhentang Zhao

**Affiliations:** Shanghai Institute of Applied Physics, Chinese Academy of Sciences, Shanghai, 201800 China

## Abstract

Generation of ultrashort coherent radiation pulses in the extreme ultraviolet (EUV) and x-ray regime is of remarkable interest in the synchrotron radiation user community. In this work, a novel technique is proposed for directly imprinting strong coherent microbunching on the electron beam with very small laser-induced energy spread. Theoretical analysis and numerical simulations demonstrated that this technique can be used for the generation of megawatt-scale level, fully temporal coherent femtosecond EUV and soft x-ray radiation pulses at a storage ring light source.

## Introduction

Evolutionary increases in storage ring (SR) source brightness over the past decades have supported a robust array of x-ray capabilities, impacting on many disciplines, such as physics, chemistry, biology and material science, etc.^1^. While synchrotron radiations as short as a few tens of picoseconds from the SR have already become a standard tool to study the structures of matters on the atomic scale, their pulse durations are still too long to measure the atomic motion and structural dynamics on the fundamental time scale of a vibrational period (~100 fs). Worth still, synchrotron radiation pulses are not suitable for some high-resolution spectroscopy and imaging experiments since they are incoherent in time.

The urgent need for intense ultrashort radiation pulses in the extreme ultraviolet (EUV) and x-ray region has prompted new developments of advanced accelerator-based light sources such as high-gain free-electron lasers (FELs). To date, several EUV and x-ray FEL facilities were constructed worldwide and have been applied to diverse cutting edge researches^2–5^. Although these high-gain FELs have met most criteria of future light sources, they usually have relatively low repetition rates and limited numbers of user beamlines. In comparison with the FEL, SR has preponderances in mature technology, high average flux, pulse-to-pulse stability and providing powerful support for a lot of users simultaneously. It is therefore worthwhile to develop new techniques to extend conventional synchrotron radiation sources toward short pulse duration.

Several methods have been developed in the last decade to improve the temporal properties of SRs^6–18^. Most of these methods employ external femtosecond lasers to manipulate the distributions of the relativistic electrons to precisely tailor the properties of the radiation pulses. One of the promising approaches is so-called “laser slicing” technique^6, 7^. It has been applied in several SRs and immediately opened up many new experimental opportunities for capturing ultrafast dynamics. However, in the laser slicing method, only a fraction of the electrons participates in the femtosecond synchrotron radiation and the radiation pulse is incoherent, thus the average flux and brightness of the source will suffer a significant loss. A substantial gain in photon flux can been achieved by using coherent harmonic generation (CHG) technique^8–10^, which borrows the idea from seeded FELs to imprint coherent microbunching on the electron beam with external laser source to significantly enhance the output intensity and improve the temporal coherence. The CHG scheme consists of two short undulators separated by a small chicane and uses electron beams as the medium to convert the seed laser to high harmonic radiation. In the first undulator (modulator), an ultrashort laser pulse is used to interact with the electrons to generate energy modulation at the scale of optical laser wavelength. Then a magnetic chicane (dispersion section) converts the formed energy modulation into an associated density modulation (micro-bunching) giving rise to a short pulse of coherent radiation at harmonics of the seed in the second undulator (radiator). The density modulation of the electron beam that contains high harmonic components can be quantified by the bunching factor. Analytically, the bunching factor of CHG is given by^9^

1 $${b}_{n}={J}_{n}(n{k}_{s}{\xi }_{c}\frac{{\rm{\Delta }}\gamma }{\gamma })\exp [-\,\frac{1}{2}{(n{k}_{s}{\xi }_{c}{\sigma }_{\gamma }/\gamma )}^{2}],$$

where *J*_*n*_ is the *n*th order first class Bessel function, *k*_*s*_ is the wave number of the seed laser, *ξ*_*c*_ is the momentum compaction of the dispersive chicane, *γ* is the relativistic parameter for the mean beam energy, Δ*γ* is the energy modulation amplitude induced by the seed laser and *σ*_*γ*_ is the slice energy spread of the electron beam. The output power of CHG is proportional to the square of *b*_*n*_, which makes it orders of magnitude brighter than that of an equivalent incoherent source.

The CHG technique has been applied to the SR to generate coherent radiation in the ultraviolet range and below^13–16^. Typically generating *n*th harmonic of the seed laser requires Δ*γ* to be approximately *n* times larger than *σ*_*γ*_. Because of the large inherent energy spread of the beam in SR, the harmonic number of a standard CHG is about one order of magnitude lower than what is required for reaching EUV and x-ray region. Further extension of the CHG output to shorter wavelength seems possible but very challenging. For example, it has been proposed that the frequency up-conversion efficiency of CHG can be enhanced by using a seed laser operating at TEM01 mode to introduce an angular modulation into the electron beam^17^. However, an intense seed laser with peak power up to terawatts level is needed for the realization of this technique and it is very challenging to operate a UV laser with the TEM01 mode. The phase-merging enhanced harmonic generation (PEHG) has also been proposed to improve the frequency up-conversion efficiency of the CHG^18^, however, a transverse-gradient undulator is typically needed in PEHG and it is found that the bunching factor of PEHG will be significantly decreased for a very small energy modulation due to the transverse-longitudinal phase space coupling during the modulation process. While decades have passed and tremendous progresses have been made for generating femtosecond radiation pulses from the SR, there is still no clear path on how to extend the wavelength of the ultrashort coherent synchrotron radiation to the EUV and x-ray region and improve the output intensity simultaneously.

We report here a novel technique to introduce strong coherent microbunching into the electron beam from the SR. It is found that sufficient large bunching factor at ultra-high harmonics of the seed can be generated by a seed laser with relatively low power. With realistic beam parameters of the SR, it shows that coherent femtosecond EUV and x-ray radiation pulses with peak power of the order of about tens MW can be produced in a short undulator.

## Results and Discussions

**Figure 1:**
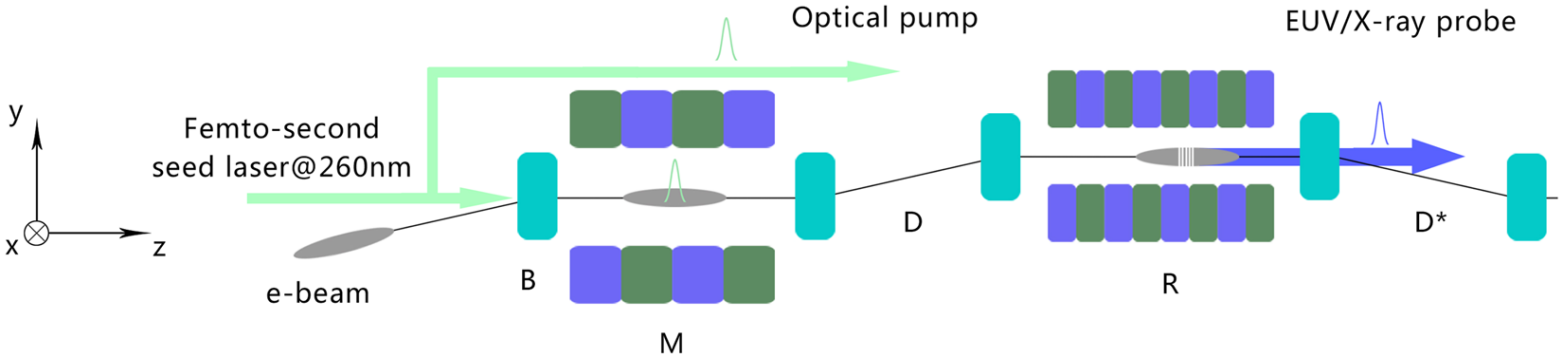
Schematic layout of the proposed scheme. The electron beam first passes through a bending magnet (B) to get an angular dispersion. This electron beam interacts with a femto-second seed laser pulse at 260 nm in the first undulator (M) to generate an energy modulation. The dogleg (D) is adopted to convert the energy modulation into coherent microbunching. Then the pre-bunched electron beam will be sent into the second undulator (R) for high harmonic generation. The second dogleg (D*) is used to compensate the transverse dispersion induced by the first dogleg. The seed laser can be split in two branches and one of them together with the EUV/X-ay radiation pulse can be applied for the femto-second pump-probe experiments at storage rings.

The layout of the proposed scheme is similar to a conventional CHG scheme (as shown in Fig. 1). However, a magnetic dipole (B) with bending angle of *b* is added upstream of the modulator, and a dogleg (D) that consists of two dipole magnets of opposite polarity is adopted instead of the chicane as the dispersion section. The first dipole is used to introduce an angular dispersion into the electron beam. Then an ultrashort UV seed laser pulse is employed to interact with the electron beam in the modulator. After that, the energy modulation is converted into density modulation by the dogleg. The dispersive properties of the first dipole and the dogleg allow, if the parameters are chosen rightly, the full compensation of the initial beam energy spread to produce very sharp micro-bunches in the electron beam. This kind of electron beam would help to initiate intense coherent radiation at very high-harmonics of the seed in the following radiator. The proposed scheme should be inserted in a long drift section of the SR and the transverse dispersions should be zero upstream of the first dipole. The transverse dispersion generated by the dogleg can be fully compensated by another reversed dogleg (D*) after the radiator. The dispersion induced by the first dipole can be corrected by another two dipoles with bending angles of −2*b* and *b* elsewhere in the ring. Since the microbunching originates from the laser-beam interaction, it is easier to facilitate precision pump-probe experiments due to intrinsic synchronization of the CHG radiation with an optical pump laser, as shown in Fig. 1.

In order to make a linear optics analysis of the proposed scheme, we adopt the beam transport matrix notation of a 6 × 6 matrix for the beam vector defined by $\vec{e}$ = (*x*, *x*′, *y*, *y*′, *z*, *δ*), where *x*, *y* and *z* are the horizontal, vertical and longitudinal coordinates, *x*′ = *dx*/*dz* and *y*′ = *dy*/*dz* are the horizontal and vertical divergences and *δ* is the relative energy deviation with respect to the reference particle^19^. Here we leave (*x*, *x*′) out for simplicity, i.e., (*y*, *y*′, *z*, *δ*) is used in the following and assuming Gaussian distributions for *y*, *y*′ and *δ*. The electron beam is first sent through a thin-lens magnet dipole whose transport matrix is

2 $${{\boldsymbol{R}}}_{B}=(\begin{array}{cccc}1 & 0 & 0 & 0\\ 0 & 1 & 0 & -b\\ b & 0 & 1 & 0\\ 0 & 0 & 0 & 1\end{array}),$$

where *b* is the bending angle of the dipole. After that, the electron interacts with the seed laser in the modulator and gets an energy change *δ*_1_ = *δ* + Δ*γ*sin(*k*_*s*_ *z*)/*γ*. Considering electrons in one seed wavelength range, the electron beam around the zero crossing of the seed laser gets an almost linear energy chirp *h* = *k*_*s*_Δ*γ*/*γ*, and the energy change of the electron can be written as *δ*_1_ ≈ *δ* + *hz*. Then the corresponding transport matrix for this part of electron beam can be derived as

3 $${{\boldsymbol{R}}}_{{\rm{M}}}=(\begin{array}{cccc}1 & {L}_{{\rm{M}}} & 0 & 0\\ 0 & 1 & 0 & 0\\ 0 & 0 & 1 & {\xi }_{M}\\ 0 & 0 & h & 1\end{array}),$$

where *L*_*M*_ is the length of the modulator and *ξ*_*M*_ is the momentum compaction generated in the undulator. The momentum compaction of the modulator is usually much smaller than that of the following dispersion section, *ξ*_*M*_ is usually ignored in a linac-based FEL. However, the energy spread in the SR is about one order of magnitude larger than that of a linac, as a result, the longitudinal phase space is more sensitive to the momentum compaction factor and *ξ*_*M*_ should be considered here to get more accurate optimum parameters for the proposed scheme. After the modulator, the energy modulation is converted into density modulation by a dogleg with transport matrix of

4 $${{\boldsymbol{R}}}_{D}=(\begin{array}{cccc}1 & {L}_{D} & 0 & \eta \\ 0 & 1 & 0 & 0\\ 0 & \eta  & 1 & {\xi }_{D}\\ 0 & 0 & 0 & 1\end{array}),$$

where *L*_*D*_ is the length of the dispersion section, *η* and *ξ*_*D*_ are, respectively, the dispersion and the momentum compaction generated in the dogleg. It is easy to show the transport matrix for the whole CHG beam line:

5 $${\boldsymbol{R}}={{\boldsymbol{R}}}_{D}\cdot {{\boldsymbol{R}}}_{M}\cdot {{\boldsymbol{R}}}_{B}=(\begin{array}{cccc}1+hb\eta  & L & h\eta  & \eta -Lb\\ 0 & 1 & 0 & -b\\ b(1+h{\xi }_{D}) & \eta  & 1+h{\xi }_{D} & \xi -\eta b\\ hb & 0 & h & 1\end{array}),$$

where *L* = *L*_*M*_ + *L*_*D*_ and *ξ* = *ξ*_*M*_ + *ξ*_*D*_ are the total length and momentum compaction of the beam line before the radiator. According to the equation (5), the longitudinal position of the electron after the density modulation can be written as

6 $${z}_{1}=b(1+h{\xi }_{D})y+\eta y^{\prime} +(1+h{\xi }_{D})z+(\xi -\eta b)\delta .$$

For a conventional CHG beamline, *b* = 0 and *η* = 0 can be satisfied, and equation (6) reduces to *z*_1_ = (1 + *hξ*_*D*_)*z* + *ξδ*. In order to generate sufficient bunching factor at very high harmonics, the optimized condition is 1 + *hξ*_*D*_ = 0 and the bunching factor is determined by the product of *ξ* and *σ*_*γ*_, as shown in equation (1). In a SR, *σ*_*γ*_ is typically on the order of a few MeV. In order to extend the harmonic number to *n* > 10, one needs to reduce *ξ* by increasing the energy modulation amplitude Δ_*γ*_. However, the required laser power scales as the square of Δ_*γ*_, which means that the seed laser power grows quickly as harmonic number increases. And the increased energy spread may perturb the nominal operation of the SR if it exceeds some threshold.

For the proposed scheme, equation (6) is changed to *z*_1_ = *ηy*′ when the optimized conditions 1 + *hξ*_*D*_ = 0 and *ξ* = *ηb* are satisfied. The high harmonic bunching factor will be determined by the product of *η* and the initial beam vertical divergence *σ*_*γ*_. A rigorous derivation of the proposed scheme gives a straightforward bunching factor as

7 $${b}_{n}={J}_{n}(n{k}_{s}{\xi }_{D}\frac{{\rm{\Delta }}\gamma }{\gamma })\exp [-\,\frac{1}{2}{(n{k}_{s}\eta {\sigma }_{y\text{'}})}^{2}].$$

In order to enhance the bunching factor for a given Δ_*γ*_ in the proposed scheme, one can increase the beta function at the entrance of the proposed scheme to reduce *σ*_*γ*_ or increase the strength of the first dipole to reduce the required *η*. Although the balance between quantum excitation and radiation damping results in a relative large energy spread in the SR, it nonetheless provides a beam with a quite low transverse emittance. The proposed technique makes full use of this feature and thus has the potential to significantly enhance the frequency up-conversion efficiency of the CHG.

Theoretical linear calculations can just give an estimation of the optimized condition for the proposed scheme, three-dimensional (3D) numerical simulations are necessary to show the possible performance of the proposed scheme. The electron beam is tracked through the beam line using the code ELEGANT^20^ with second-order transport effects taken into account. The energy modulation and the FEL lasing processes were simulated using the time-depended mode of GENESIS^21^. These simulations considered realistic transfer matrixes of the elements in the proposed scheme. Various nonlinear effects such as coherent synchrotron radiation and incoherent synchrotron radiation are also included in these simulations. We use the nominal parameters of a 3^rd^ generation SR and a diffraction-limited SR as representative examples. The electron beam parameters used in the simulations are listed in Table 1. In order to enhance the bunching factor at ultra-high harmonics, we assume that the geometric vertical emittance is about 2 pm. The rms intrinsic vertical divergence and beam size are assumed to be about *σ*_*γ*_ = 0.2 *μ*rad and *σ*_*γ*_ = 100 *μ*m, respectively, at the entrance of the proposed scheme.

**Table 1:** Nominal electron beam parameters used in the 3D simulations.

Beam energy	1.5 GeV
Relative energy spread	0.1%
Peak current (*I*_0_)	300 A
Geometric horizontal emittance	2 nm rad/0.2 nm rad
Geometric vertical emittance	2 pm rad

For comparison purpose, simulations have been performed for both the 3^rd^ generation and diffraction-limited SR light sources.

**Figure 2:**
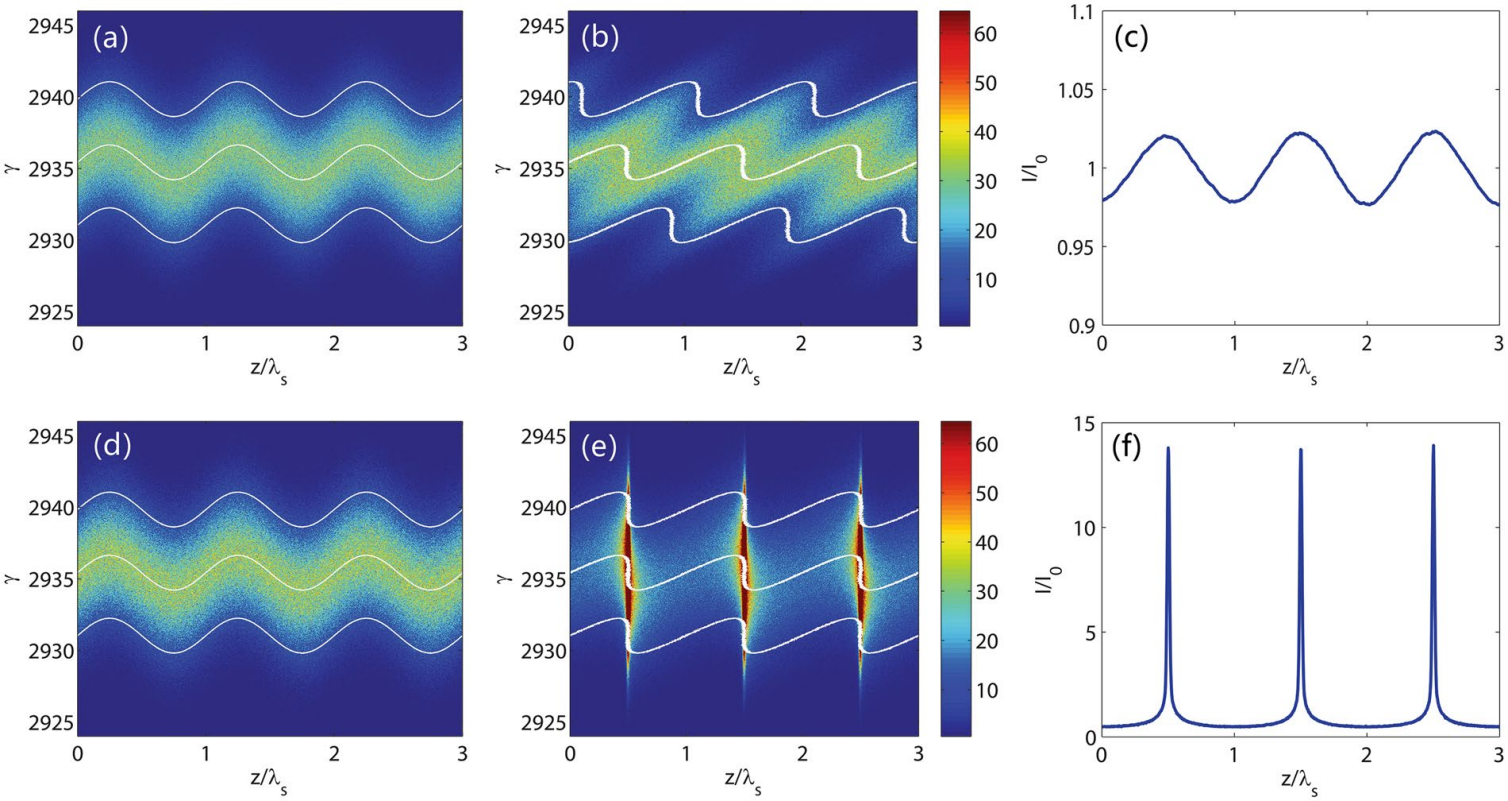
Comparison of the mechanisms of the conventional CHG and the proposed scheme. Longitudinal phase space distributions at the exits of the modulator (**a**) and the dispersion section (**b**) and the corresponding current distribution (**c**) in the conventional CHG. Longitudinal phase space distributions at the exits of the modulator (**d**) and the dispersion section (**e**) and the corresponding current distribution (**f**) in the proposed scheme. Three electron slices have been highlighted to show the principle. With a very small energy modulation of about 0.4 times of the initial beam energy spread, sharp coherent mocrobunchings can be generated via the proposed scheme. The corresponding bunching factor is very high for high harmonics.

**Figure 3:**
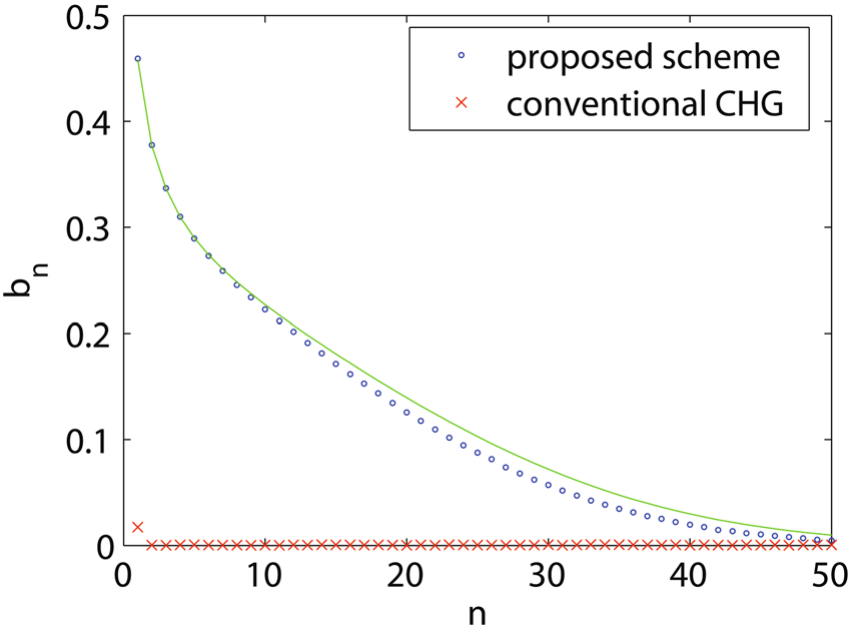
Comparison of the bunching factors of the conventional CHG and the proposed scheme. The red crosses are the results from 3D simulation for the conventional CHG with an energy modulation amplitude of about 0.4 times of the initial beam energy. The green solid curve is the result from the theoretical calculation and the blue dots are the results from 3D simulations for the proposed scheme.

After passing through a dipole with a length of 0.1 m and a bending angle of about 10 mrad, the electron beam is sent into a short modulator with a period length of 8 cm and a period number of 10 to interact with seed laser pulse at 260 nm. The seed laser power profile is assumed to be Gaussian with a pulse length of 30 fs (FWHM) and a peak power of about 200 MW. The laser waist is assumed to be 1 mm. The dipole magnet in the dispersion section has a length of 0.1 m and a bending angle of about 10 mrad. The distance between two dipoles in the dispersion section is 0.85 m. For comparison purpose, we carried out 3D simulations for both conventional CHG and the proposed scheme. The simulation results are summarized in Figs 2 and 3. In the proposed scheme, the first dipole creates a correlation between the angular divergence and energy deviation of the electron beam. As the initial angular divergence of the electron beam is very small, the angular divergence represents the electron energy deviation after passing through the first dipole. Here we choose three slices in beam energy (indicated in white) to show the evolution of the longitudinal phase space. These three slices also have different *y*′ due to the correlation. The energy modulation amplitude induced by the seed laser is about 0.6 MeV, which is much smaller than the intrinsic beam energy spread (1.5 MeV). One can find in equation (4) that the change of the longitudinal position of the electron after passage through the dogleg is Δ*z* = *ηy*′ + *ξδ*. For a conventional CHG with a chicane as the dispersion section (*η* = 0), the micro-bunches in each slice will be shifted apart due to the different energy (Fig. 2b) and results in a weak bunching (Fig. 2c). However, noticing that these slices also have different *y*′, thus we can tune the *η* of the dogleg to fully compensate the energy spread effect (Δ*z* = 0) and get very sharp micro-bunches around the zero-crossing phase of the seed, as shown in Fig. 2e,f. Figure 3 shows the bunching factor at various harmonic numbers at the exit of the dispersion section for the conventional CHG and the proposed scheme. For conventional CHG scheme, one can only get a bunching factor of about 0.02 at the fundamental wavelength due to the very small energy modulation. While for the proposed scheme, one can find that the bunching factor is about 0.13 at the 20^th^ harmonic and is about 0.06 at the 30^th^ harmonic. These bunching factors are sufficiently large to effectively suppress the electron beam shot noise and to generate coherent radiation in the following radiator. For small harmonic numbers, the bunching factor distribution from simulations fits quite well with the one-dimensional analytical results which are directly calculated by equation (7). However, for harmonic number larger than 20, the deviation becomes prominent mainly due to the second order transport effects. We also found with 3D simulations that the second order effects will significantly decrease the bunching factor when the harmonic number is larger than 100, and the output wavelength will eventually limited to about 1 nm for the proposed scheme.

**Figure 4:**
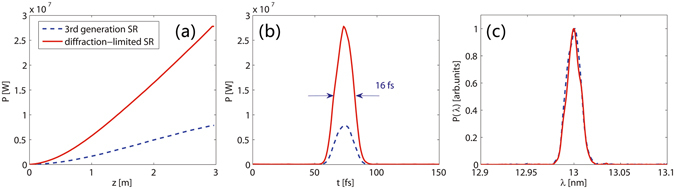
FEL performance of the proposed scheme. Simulations have been performed with parameters of a 3^rd^ generation SR (blue line) and a diffraction limited SR (red line): (**a**) peak power growth as a function of the radiator distance; (**b**) output radiation pulses and (**c**) output spectra at the exit of the radiator.

Here we adopt a 3 m long radiator with a period length of 3 cm to generate coherent radiation pulses at 13 nm with the proposed method. The simulation results are illustrated in Fig. 4. Simulations were performed with the electron beam parameters from both 3^rd^ generation and diffraction-limited SRs. The large bunching factor offered by the strong microbunching is responsible for the initially steep quadratic power growth in Fig. 4a. The significant enhancement in FEL performance is clearly seen. After a 3 m long radiator section, the output peak power in the 3^rd^ generation SR case is about 7.8 MW (~10^10^ photons/pulse). In the diffraction-limited SR case, the radiation peak power exceeds 27 MW at the exit of the radiator. Due to the harmonic pulse shortening effect^22^, the output pulse durations in these two cases are both around 16 fs (FWHM). From Fig. 4c, one can find that the output spectral bandwidths are about 0.13% (FWHM), which is close to the Fourier-transform limit.

**Figure 5:**
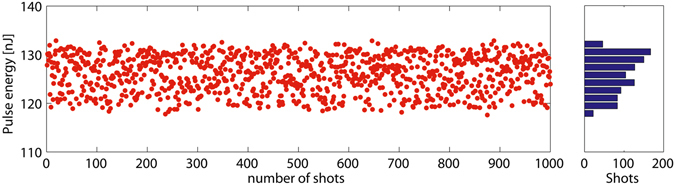
Output stability of the proposed scheme. Jitter of the radiation pulse energy at various shots for fluctuating amplitudes of the laser power and magnetic fields of the dipoles.

The performance of the proposed technique may be affected by some practical issues such as the fluctuation of the seed laser power and the instabilities of the magnetic fields of the dipoles. Here we choose 5% (rms) and 0.1% (rms) for the criteria of the laser power and the magnetic field stabilities, respectively, which are feasible with state-of-the-art commercial laser and accelerator technologies. The resulting fluctuations of the radiation pulse energy at the exit of the radiator for the 3^rd^ generation SR case are shown in Fig. 5. One can find that the pulse energy of about 120 nJ can be well maintained, which demonstrates the stability of the proposed scheme.

## Conclusions

In conclusion, a novel technique is proposed to generate stable, coherent and ultra-short EUV and x-ray radiation pulses with high intensity by fully using the advantage of the electron beam from a SR. This kind of light source would allow one to perform many challenging experiments which require ultrafast and narrow-bandwidth radiation pulses. One unique advantage of the proposed scheme is that it doesn’t require high power seed lasers, which would allow the use of a laser with much higher repetition rate compared to the conventional CHG. Furthermore, the small modulation amplitude makes it possible to reuse the same bunch at a high rate without blowing up its energy spread. In addition to SR, the proposed scheme has other potential applications involving the use of electron beam with large intrinsic energy spread from laser-plasma accelerators. And it can also be used to enhance the frequency up-conversion efficiency of a seeded high gain FEL with the help of the flat-beam technique^23^ or improve the performance of the enhanced SASE^24^ with a relative small laser induced energy spread. Further investigations on these topics are ongoing.
